# Analyzing University Students’ Perceptions Regarding Mainstream Pornography and Its Link to SDG5

**DOI:** 10.3390/ijerph19138055

**Published:** 2022-06-30

**Authors:** Itsaso Biota, Maria Dosil-Santamaria, Nahia Idoiaga Mondragon, Naiara Ozamiz-Etxebarria

**Affiliations:** 1 Department of Education Sciences, University of the Basque Country UPV/EHU, 48940 Leioa, Spain; itsaso.biota@ehu.eus; 2 Department of Research and Diagnostic Methods in Education, University of the Basque Country UPV/EHU, 48940 Leioa, Spain; maria.dosil@ehu.eus; 3 Department of Developmental and Educational Psychology, University of the Basque Country UPV/EHU, 48940 Leioa, Spain; nahia.idoiaga@ehu.eus

**Keywords:** sex education, pornography, sustainability

## Abstract

Background: Violence against women and girls continues to be a widespread problem, and its elimination is one of the targets of the Sustainable Development Goal 5 toward achieving gender equality. One of the main causes of this violence is the structural sexism present in societies that continues to be perpetuated through pornography, especially among young people. Therefore, the main objective of this research is to analyze the consumption of pornography among young people, studying its effects and relationship with affective-sexual education. Methods: This study was carried out with a sample of 280 students in the north of Spain. The ages of the entire sample ranged from 18 to 37 years (*M* = 20.3, *SD* = 2.6). From the sample, 78.9% (*n* = 221) define their gender identity as women and 21.1% (*n* = 59) define their gender identity as men; no person within this research self-identify as non-binary. The instrument used was the Survey on Affective-sexual Education and Pornography (Ballester et al., 2019). The sample was recruited by snowball sampling. Results: The results of the present study show that the average age at which people start watching pornography is 10.4 years. The majority of young people consume it to satisfy their curiosity. In addition, with regard to gender, boys consume more pornography, especially for masturbation. Finally, 20.5% of the participants believe that the sexual education they have received has not been adequate, and most of them solve their sexual doubts by asking friends. Conclusions: The results indicate that it is necessary to improve the affective-sexual education provided in schools, since students consume pornography at a very early age, and young people have normalized its consumption to address their curiosity and satisfy their sexual needs. Finally, the impact of pornography consumption on SDG5 was reflected on.

## 1. Introduction

### 1.1. Mainstream Pornography and Gender-Based Violence

In recent decades, due to the rise of the use of the Internet and easy access to new technologies, the production and consumption of pornography have increased exponentially [1]. Today, pornography is a macro-industry that generates millions of dollars in profits annually [2]. As a result, online pornography websites are among the most visited of 2021, and specifically, the pornography platforms “XVideos” and “Pornhub” rank 9th and 11th, respectively [3].

The type of pornography that can be found on these portals—also called mainstream pornography—is characterized by a clear focus on satisfying men’s desires, where a hegemony of male power relations over females can be observed [4,5,6,7]. Therefore, several researchers and experts have pointed out that pornography is associated with gender inequality, sexism, and violence against women [8,9]. Furthermore, many scenes encourage the eroticization of violence toward women, since many of the videos contain sexual practices in which women are raped or represented as passive objects devoid of desire [10,11]. In addition, mainstream pornography may contain videos with incestuous themes and can even be related to pedophilia, including videos in which childlike actors and actresses have sex with their parents or teachers [12]. Thus, the topics that users can find through searches on these webpages range from the word “incest” to “teen raped” [13].

#### 1.1.1. Types of Pornography and Its Consumption by Age and Gender

Concerning the age at which users start consuming pornographic material, a recent study conducted in Spain by Ballester, Orte, and the Youth and Inclusion Network [14] concludes that the age at which users start consuming pornography is currently eight years old. Aside from this finding, it is also worth highlighting the results related to gender, as these observed that men start earlier than women, with 70–80% of men starting before the age of 16 and 48% of women at older ages, between 16 and 18. As a result, many minors—especially men—are currently accessing pornographic content without sufficient maturity and knowledge on affectivity and sexuality.

Regarding the type of pornographic content accessed by these young people, research indicates that mainstream pornography contains practices that, in most cases, are unrealistic and even dangerous for people’s health and integrity, especially for women [15]. In a study conducted by Bridges et al. [16], the results indicated high levels of aggression and violence in pornography—both verbal and physical. From the 304 scenes analyzed, 88.2% contained physical aggression, mainly spanking, gagging, and slapping, while 48.7% of the scenes contained verbal aggression, mainly insults. The perpetrators of the assaults were usually men, while the victims were mostly women. The victims showed pleasure or responded neutrally to the assault most of the time.

The high presence of violence in pornography has resulted in what Alario [10] calls the “eroticization of violence against women”, which was categorized by the author into six types of eroticization perpetuated by the pornography industry: the eroticization of women’s physical pain, lack of desire, suffering, humiliation, sexual abuse of minors and the eroticization of prostitution.

#### 1.1.2. Main Effects of Mainstream Pornography Viewing

Regarding the effects of pornography viewing, a meta-analysis by Weight et al. [17] found associations between watching pornography and violent behavior. According to this study, men who consume pornography are more likely to practice or desire dominant and degrading practices, such as gagging and choking. They also found that women who consume pornography are more likely to engage in or desire submissive practices. According to this meta-analysis, a longitudinal study among adolescents revealed that watching pornography is associated with sexually violent behavior across the lifespan.

Yvarra et al. [18] conducted the aforementioned longitudinal study, which examined longitudinal links between intentional exposure to pornographic material and sexually aggressive behavior among 10–15-year-olds surveyed nationally in the United States. The study found that intentional exposure to violent, pornographic material over time predicted a nearly 6-fold increase in the odds of self-reported sexually aggressive behavior.

In addition to lacking any affectivity, pornography depicts scenes based on a state of subjugation of women, fostering and perpetuating gender inequality and transmitting stereotypes and macho roles [17]. Moreover, the frequent and continued consumption of this type of pornography can have consequences for the youngest members of society, given that the younger the age of exposure to pornography, the greater its impact on behavior, self-esteem, and sexual habits when reaching adulthood [19].

According to a University of Nebraska study conducted by Bischmann et al. [20], the age of a child’s first exposure to pornography may be associated with certain sexist attitudes developed throughout life. The research, conducted with an all-male sample, found that the younger a man at his first exposure to pornography, the more likely he is to desire power over women. In addition, the study concluded that pornography viewing has a “real” impact on heterosexual men, particularly in terms of their perceptions of gender roles.

On the other hand, pornography may generate unattainable expectations within a couple and the desire to engage in practices that may become violent [21]. In a study by Ballester et al. [22], it was pointed out that one of the worst effects of pornography consumption in adolescents and young people was perceptual distortion, the formation or deformation of attitudes, given that due to the evolutionary stage in which they find themselves, they do not have the emotional maturity to understand what they see.

Collazo (2019) [23] argues that the scientific literature indicates some people have difficulties with sexual behavior due to what is visualized in pornographic scenes. Furthermore, as Nathawat et al. [24] point out, pornography allows for the possibility to experiment from a position of curiosity, becoming less normative and enabling practices that are considered crimes, such as rape.

#### 1.1.3. The Pornification of Culture

Consequently, societies face a phenomenon known as the “pornification of culture”, where sex industries, such as pornography, have an increasing influence and significance [8]. According to Favaro et al. [25], this is a Western phenomenon aimed at spreading the idea that pornography is inevitable and that it can also be a positive characteristic of living a good life. However, considering the nature and characteristics of the “new pornography” and given the normalization of pornography consumption, one of the most concerning realities that we must bear in mind “is that the narratives and values of these industries penetrate every day, reconfiguring the sensibilities, subjectivities and sexual practices of the majority” [25]. Likewise, the scope of current consumption has also become what has, in recent feminist debates, become known as “rape culture” [26]. According to De Miguel [27], this is “a culture in which it is possible to enjoy everything that one would not tolerate doing to women in real life: insulting them, pulling their hair, penetrating them in groups and in every possible way, enjoying their stupefied and suffering face, and eroticizing contempt and violence”. In other words, through the eroticization of violence, the physical and power abuse of women is socially legitimized, which is something that is of particular concern to contemporary societies and one of the central themes of the 2030 agenda and the Sustainable Development Goals.

### 1.2. The 2030 Agenda, SDG 5 and Pornography Consumption

In 2015, The United Nations (UN) adopted the 2030 Agenda for Sustainable Development (SD) with the 17 Sustainable Development Goals (SDGs), which all UN member states have committed to achieving by 2030. Those SDGs were designed to foster sustainable development across three key areas: economic, social, and environmental, and seek to achieve gender equality and the empowerment of all women and women to achieve human rights for all [28].

Undoubtedly, gender equality is embedded in every SDG, and it is considered a catalytic policy intervention and one of the essential foundations for building a prosperous and sustainable world [29]. Furthermore, the empowerment of women and women will make a crucial contribution to progress across all the goals and targets, since the achievement of full human potential and sustainable development is not possible if one-half of humanity continues to be denied full human rights and opportunities [28].

The SDG framework has a specific goal regarding Gender Equality (SDG5—Achieve gender equality and empower all women and girls). To explore the realization of this goal, the 2019 SDG Gender Index provides country-level data measuring the state of gender equality aligned to 14 of the 17 SDGs [30]. The 2019 SDG Gender Index presents a challenging picture and finds that globally, nearly 40% of girls and women live in countries failing on gender equality. With 79.7 points out of 100, Spain is ranked 23rd out of 129 countries. On the other hand, it obtains relatively good indexes in the 4th goal of education related to gender, and in the 5th goal of Gender Equality itself, it scores 90.2 out of 100 [30].

Although progress has been made, and the overall SDG 5 score seems to provide reason for optimism, there is still a long way to go to achieve real equality [31]. In fact, the second target of SDG5 is “to eliminate all forms of violence against all women and girls in public and private spheres, including trafficking and sexual and other types of exploitation” [28]. Although many advances and achievements have been made in recent decades regarding equality, women and girls worldwide continue to suffer serious inequalities, situations of vulnerability, and sexual, economic, physical, and psychological violence throughout their life cycle [32].

In this context, it should be emphasized that currently, scientific evidence has found that pornography [10,22,25] constitutes channels through which sexist models that reproduce inequality, discrimination and violence toward women are transmitted. In fact, pornography constitutes one of the most sexist and violent spaces and means of socialization toward women, being a clear obstacle to development [33].

### 1.3. The Present Study

Thus, considering the consequences that the consumption of pornography can have, both at the individual level but also for the development of societies, the present study aims to analyze the consumption of pornography in a retrospective way within a sample of young university students in the north of Spain. Even though research already indicates that consumption of pornography begins at approximately eight years of age [34], researching with minors being a sensitive issue, the study was conducted with a university population, assuming the cohort’s sufficiently mature stage to cope with the survey and also to describe their consumption story from a retrospective viewpoint until present.. Students were asked about their use of pornography in their early teenage years and youth and also about the affective-sexual education they have received during their recent youth. Another aim is to determine when they started using pornography, their motives and the possible effects of continued pornography use. To this end, participants were asked about both current pornography use and pornography use in the last ten years.

## 2. Materials and Methods

### 2.1. Design

This is a quantitative method with a descriptive and correlational cross-sectional design. The sample inclusion criteria were: to be of legal age and to be a university student. The sample was a convenience sample, and recruitment was indirect.

### 2.2. Participants

This study was carried out with 280 students from the University of the Basque Country/Euskal Herriko Unibertsitatea (UPV/EHU). The age of the whole sample ranged from 18 years to 37 years (M = 20.3, SD = 2.6). From the sample, 78.9% (*n* = 221) identify with a female gender identity, and 21.1% (*n* = 59) identify as male, and no person in this survey identified as non-binary.

Regarding sexual orientation, 66.4% (*n* = 186) identify as heterosexual, 25.4% (*n* = 71) as bisexual, 5% (*n* = 14) as homosexual, 2.5% (*n* = 7) as undefined, and 0.7% (*n* = 2) did not answer. Concerning residential status, 90.3% (*n* = 251) of the sample resided in the Basque Autonomous Community (BAC) and 9.7% (*n* = 29) resided outside the BAC. Regarding current activity, 56.4% (*n* = 158) were only studying, 26.1% (*n* = 73) were working and studying, 11.4% (*n* = 32) were looking for work while studying and 5.7% (*n* = 17) of the sample did not answer this question. Most participants lived with their parents, 82.1% (*n* = 230), 7.5% (*n* = 21) lived in shared apartments with other people, 5% (*n* = 14) lived in other situations, 3.2% (*n* = 9) lived with their partners, and 2.1% (*n* = 6) lived alone.

### 2.3. Procedure and Instrument

First, an online questionnaire was created in Google Forms and sent via social networks. The questionnaire explains both the objectives of the study and the procedures to be followed during the study as well as the right to voluntary withdraw from the study if decided afterwards. All standards established by Organic Law 15/99 on Personal Data Protection were followed for data collection.

The questionnaire involved two parts. Firstly, participants had to answer an ad hoc sociodemographic questionnaire about age, sexual identity, sexual orientation, current activity, current studies, life situation, feminism, and an analysis of the effect of pornography on sexism. The estimated time to complete this questionnaire was two minutes.

Secondly, participants were asked to complete the Survey on Affective-sexual Education and Pornography [34]. This survey has been validated both empirically and by judges, and it is a scale based on a sociological survey. The survey is composed of two dimensions. The first dimension collects background information on affective-sex education and pornography use in the last ten years, while the second dimension focuses on current use. Some items of the scale include “have you received affective-sexual education in the last ten years”, “in these ten years, did you watched pornography”, “why did you watch pornography these last ten years”, “do you watch pornography”, and “how often do you watch pornography”. The total number of items is 33, and the estimated time for completing the set of the two dimensions is 10 min.

### 2.4. Data Analysis

Data were analyzed using IBM SPSS Statistics for Windows, Version 26.0 (Armonk, Westchester, NY, USA). Firstly, the frequencies and percentages of the sociodemographic variables were described to present the profile of the participants. Subsequently, analyses were conducted between the variables subject to study to explore significant associations between the perception of affective sexual education and pornography in two viewing temporal dimensions (last ten years and present) as a function of gender and age.

## 3. Results

### 3.1. Watching Pornography and Its Association with Other Variables

The average age at which participants started watching pornography for the first time was 10.4 years old, with the first images of pornography being consumed at the age of 11.3 years. Table 1 shows the reasons for currently watching pornography according to gender, showing a statistically significant association with a large effect size (*x*^2^ (5) = 71.66, *p* = 0.001, *Vcramer =* 0.51). In addition, the use of pornography for masturbation is significantly higher in men than women, with 46% of men in the sample reporting watching pornography for this reason. However, there were no statistically significant associations between age and this item.

### 3.2. Frequency of Pornography Watching according to Temporal Dimension, Gender, and Age

Table 2 shows the statistically significant association between the frequency at which young people watched pornography in the last ten years according to gender. This association is statistically significant, *x*^2^ (3) = 58.68, *p* = 0.001, *Vcramer* = 0.46, with an intermediate effect size. Women show higher scores than men except on the items ‘never’ and ‘no response’. The present time item also showed a statistically significant association with gender, with a large effect size, *x*^2^ (3) = 105.62, *p* = 0.001, *Vcramer* = 0.61. The trend is repeated, with men showing a higher frequency than women. Table 3 also shows that the younger age group has a significantly higher association than the older age group in watching pornography sometimes for both times, in the last 10 years *x^2^* (3) = 12.45, *p* = 0.014, *Vcramer* = 0.21, and at present, *x*^2^ (3) = 10.85, *p* = 0.028, *Vcramer* = 0.20.

### 3.3. Pornography Watching Context according to Temporal Dimension, Gender, and Age

There are statistically significant differences in the context of viewing pornography (alone or accompanied) according to gender for the two temporal dimensions, the last 10 years, *x*^2^ (3) = 27.34, *p* = 0.001, *Vcramer =* 0.31, and the present, *x*^2^ (2) = 55.90, *p* = 0.001, *Vcramer =* 0.45. The majority of young people indicate they have viewed pornography alone. In the case of the last ten years, 46.4% (*n* = 130) of the women and 20% (*n* = 56) of the men showed this trend. The same pattern appeared for the present time; 28.6% (*n* = 80) of the women and 18.6% (*n* = 50) of the men watch pornography alone. As a function of age, no statistically significant associations are shown with any companionship items for watching pornography.

### 3.4. Positive and Negative Effects of Pornography as a Function of Temporal Dimension, Gender, and Age

The most valued positive effects on the participants’ experience differ significantly according to gender at both temporal dimensions; that is, in the last ten years, *x*^2^ (4) = 50.66, *p* = 0.001, *Vcramer =* 0.43 and currently, *x*^2^ (4) = 58.55, *p* = 0.001, *Vcramer =* 0.46, both with a moderate effect size (see Table 4). Women show a higher percentage except in the item about facilitating masturbation, where men show a higher frequency than women both in the last ten years and currently.

No statistically significant differences are shown between the positive effect items in the last ten years according to age. However, statistically significant differences were found in the present time (currently) dimension, *x*^2^ (3) = 14.16, *p* = 0.027, *Vcramer* = 0.22. 17.9% (*n* = 50) of 18–21-year-olds and 8.6% (*n* = 24) use pornography to masturbate and 12.9% (*n* = 36) of 18–21-year-olds and 3.2% (*n* = 9) use it to socialize with friends.

Regarding the negative effects, Table 5 shows the statistically significant differences between the negative effects that affected them the most according to gender. In the case of the last ten years, *x*^2^ (4) = 36.88, *p* = 0.001, *Vcramer* = 0.36, a small effect size was found, and in the case of present time, *x*^2^ (4) *=* 50.06, *p* = 0.001, *Vcramer* = 0.42, an intermediate effect size was found.

There is a statistically significant association between the negative effects of watching pornography currently and age, *x*^2^ (4) = 14.60, *p* = 0.006, *Vcramer* = 0.22. That is, there are differences in the negative effects of watching pornography according to age. For example, 42.1% of 18–21-year-old young people (*n* = 118) indicated that watching porn affected their relationship with their parents, while among those over 22 years of age, only the following checked this option: 6.8% (*n* = 19). Likewise, in the youngest age group (18–21), 9.6% (*n* = 27) indicated that they spent too much time watching pornography, while among those over 22 years of age, only 1.4% (*n* = 4) indicated this effect.

### 3.5. Porn Addiction as a Function of Temporal Dimension, Gender, and Age

Table 6 shows the statistically significant association in self-evaluation of porn addiction according to gender. Both in the last 10 years, *x*^2^ (2) = 38.33, *p* = 0.001, *Vcramer* = 0.37, and in the present time, *x*^2^ (2) = 34.77, *p* = 0.001, *Vcramer* = 0.35, had a small effect size. Men show a higher frequency on items related to a small addiction. As a function of age, no statistically significant associations are shown.

### 3.6. Receiving and Participating in Effective Education to Resolve Sexual Questions

Regarding affective-sexual education, 71.8% (*n* = 201) of the participants indicated that they had received it compared to 28.2% (*n* = 79) who responded that they had never received it. Of those who had received affective-sexual education, 88.3% indicated that they had received it at school, 9.1% had received it in other organizations-associations, and 2.6% had received it in community services. Likewise, 54.5% of the participants indicated that their questions were partially answered in this type of educational program, 25.9% were not answered, and 20.5% stated that all their questions were resolved. Regarding the question of which other people answered the questions that arose during childhood, adolescence, and youth, 49.6% indicated that it was their friends, 20% indicated that it was the Internet, 17.1% indicated that it was their parents, and 13.2% indicated that it was their teachers.

### 3.7. Reception of Sexual Offers Related to Pornography According to Gender

Statistically significant differences were found between the receipt of sexual offers related to pornography according to gender. In fact, 45.6% of women and 10% of men point that they have received some type of sexual offer related to pornography.

## 4. Discussion

This study expands the existing information on young people’s pornography consumption by analyzing its retrospective from adolescence to young adulthood. This will lead us to discuss in the following lines not only the relationship of these young people with pornography or sexual-affective education but also the implications that this consumption may have related to SDG5. In this study, the average starting age of pornography consumption was 10.4 years, which is similar to other studies [22]. This is a worrying starting point, since children are still at an age when they have not even started adolescence. Starting so early to consume pornography can seriously affect the perception of affective-sexual relationships, as they will generalize and normalize what they see in these videos. [17]. That is, the input they receive from the mainstream pornography is a fictitious sexual-affective model based on misogyny and sexism [11]. Consequently, through watching pornography, they normalize violent sexual practices specially against women, which is something that will directly hinder the achievement of SDG5.

It will therefore be key to provide young people with tools from an early age to understand affective-sexual relationships and their idiosyncrasy through affective-sexual education. In fact, it could be positively assessed that most of the students surveyed (88%) have received affective-sexual education and that this has been provided in their schools. However, it should be noted that [35] only 20.5% believe that this education responded to their questions, curiosity and interests.

Moreover, it should also be highlighted that only 17.1% of young people have asked their parents to clarify doubts about affective-sexual relationships. This is a worrying fact because although it is common at this age for peers to be the most influential people for teenagers [36], they are often not a reliable source nor do they have sufficient training to resolve other people’s questions. Furthermore, perhaps even more alarming is that 20% of the respondents clarified their doubts through the Internet (where pornographic content is also included). This is worrying, since the Internet contains false information and pornographic content that is not regulated and usually has high sexist and biased information [16]. This can lead users, especially young people, to generate unrealistic, biased, and sexist expectations about sexual activities and normalize violent situations on a sexual level [35].

Therefore, if we really want to plan for SDG5 as an achievable objective, we should start by building quality sexual and emotional education that really reaches students. In fact, quality education is key to ensuring the acquisition of competences on gender equality and women’s empowerment and thus building a prosperous and sustainable world [29]. To this purpose, it will also be essential to analyze why these young people consume pornography and whether these reasons change over the years or for gender reasons.

Most participants of this research reported consuming pornography to satisfy their curiosity, women being most likely to consume it for this reason, which is followed by learning about sex, to masturbate, and to imitate friends. Therefore, it is notable that most young people turn to pornography without the influence of friends. Mainly they consume it to satisfy their curiosity and to learn about sex, although previous studies have already pointed out that it is a source strongly discouraged for this purpose [20].

As expected, men show the highest rates of consumption [37], particularly when they were minors. Furthermore, in all items related to porn addiction, men show a higher frequency than women in this study. In contrast, women consume pornography alone to a greater extent than men. This context could be explained because pornography is frowned upon by society, and more so among women, where both the pleasure of sex and the consumption of pornography have been taboo for many years [2].

When young people were asked about the positive effects of consuming pornography, its use for masturbation was the principal response. However, despite the fact that young people indicate that masturbation is a positive effect of pornography consumption, we should point out that this factor is something that should be worked on, so that they can be aware of the type of sexual content they are using to masturbate. Certainly, the content offered by mainstream pornography websites today is full of both explicit and symbolic violence against women through scenes in which women are subjected to violence while they are showing pleasure [16].

So, needing pornography to masturbate can lead them to feel sexual desire for these practices in which men exercise power over women [17], which can generate inequality and even gender-based violence in their current and future relationships. Moreover, given the current normalization of this type of pornography consumption among young people, some researchers point out that normalization has become a form of eroticization of violence toward women [10].

Regarding the perception of participants about the negative effects of pornography consumption, the majority of respondents said that they spent too much time consuming it. This could be because pornography consumption can create addiction, as many researchers have already warned [38]. The second more frequently mentioned negative effect is that pornography can impact couple relationships. Many young people may feel that their relationships were disturbed by the perception that they should repeat in their real life the things that they have previously seen in pornographic content [21].

This study also found significant gender differences in the receipt of pornography-related sexual offers. Of the women surveyed, 45.6%—compared to 10% of men—state that they have received some type of sexual offer related to pornography. This situation reveals the exposure and vulnerability to which women are subjected by new recruitment channels used to encourage participation in the pornographic industry. These new methods of recruiting women are on the rise (such as the famous OnlyFans platform) due to easy and massive access to the Internet [39]. This situation raises concerns about the vulnerability of many young women who could be recruited out of economic necessity to participate in so-called “sex work”.

This finding also reveals that young women’s bodies are still considered merchandise by a neoliberal society, as outlined in SDG5. Therefore, if this goal is to be achieved, it will be essential that children understand, analyze and problematize sexuality and question the interests that the sex industry has over women’s bodies from a young age. Otherwise, we will continue to perpetuate an unequal society, where ineffective sex education and widespread consumption of violent pornography will continue to increase inequalities and gender-based violence, therefore failing to achieve SDG5.

As for the limitation of the study, the generalizability of the results is constrained, since it is a non-probabilistic sample in which there may be some selection bias. Future studies should expand the sample and extend it to more countries.

## 5. Implications

The main implication of this study is that it shows the consumption of pornography among adolescents and young people is still an issue to be addressed. Of particular concern is the early age at which they start consuming pornography, its link to sexual offers received especially by women, the addiction it can create especially in underage children, the generalization of violent and misogynistic practices to real life and above all the lack of tools that young people acknowledge they have to deal with it. All of these behaviors can seriously damage the mental health of young people, and they are at a very vulnerable age where future mental health problems need to be prevented. Therefore, this is an issue that needs to be addressed through quality affective-sexual education.

In fact, in Spain (where this research has been carried out), this problem has awakened social and political interest, since the legal basis has been established to respond to this need in current Law on the Comprehensive Guarantee of Sexual Freedom [40]. Specifically, this law has regulated the implementation of affective-sexual education in compulsory education. This law postulates that the model of affective-sex education that has been adopted will be oriented toward learning how to prevent and avoid all forms of sexual violence and discrimination. According to this new law, the educational administrations shall promote the application, permanent updating and dissemination of protocols containing action guidelines for the prevention, detection and eradication of sexual violence in the educational sphere, both public and private, and for each of the educational levels. This organic law is also related to the commitments and goals of the State Pact against gender-based violence as well as the 2030 Agenda in several areas. This is important, because only a society that educates in respect and equality will be able to eradicate violence against women and girls.

With regard to pornography, in Spain, there has been a law in place since 2021 aimed at protecting children and adolescents from sexual violence [41]. This Spanish law states that public administrations will develop education, awareness and dissemination campaigns addressed to children and adolescents, families, educators and other professionals who work regularly with minors. The main objective of this program is to promote a responsible and safe use of the Internet as well as to raise awareness of the risks derived from inappropriate use that can generate sexual violence against children and adolescents, such as the consumption of pornography among minors. To these principles and proposals, the new law on Sexual Freedom in Spain has added the proposal to carry out awareness-raising campaigns specifically targeting men, adolescents and children in order to eradicate prejudices based on stereotyped gender roles as well as to actively contribute to the prevention of the demand for sexual exploitation and pornography that naturalizes sexual violence.

From this research, we would add to the points mentioned above the necessity to work toward that quality affective-sexual education from a holistic perspective. Specifically, to respond to the needs found in this study, this model should be liberating, critical, emancipatory, favoring self-knowledge of the body, and rooted in a gender perspective. Since, as we have seen, the consumption of pornography, the reasons for it and the implications of it vary by both the age and gender of the young people.

## 6. Conclusions

In conclusion, it is necessary to start working on affective-sexual education in a holistic way from childhood in order to reach the youngest, favoring critical thinking that will help them avoid the influences of mainstream androcentric pornography. By doing so, young people will receive an education that will help them to fully develop their affective-sexual health, which will be key to meeting the SDGs and fulfilling the 2030 agenda as a society.

## Figures and Tables

**Table 1:** Reasons for Currently Watching Pornography according to Gender.

Reasons for Current Use of Pornography	Gender	*n* (%)	*x*^2^ (*p*)	*Vcramer*
To satisfy curiosity	women men	10 (3.6%) 4 (1.4%)	71.66 (0.001 ***)	0.51
To learn about sex	women men	8 (2.9%) 6 (2.1%)		
To masturbate	women men	40 (14.3%) 45 (16%)		
Because my friends did it	women men	1 (0.4%) 1 (0.4%)		
For other reasons	women men	3 (1.1%) 2 (0.7%)		
No response	women men	154 (55%) 6 (2.1%)		

Note: *** *p* < 0.001.

**Table 2:** Frequency of Pornography watching in the last 10 years according to Gender.

Frequency of Watching Pornography	Gender	*n* (%)	*x*^2^ (*p*)	*Vcramer*
Sometimes (last ten years)	women men	124 (44.3%) 30 (10.7%)	58.68 (0.001 ***)	0.46
Sometimes (current)	women men	67 (23.9%) 19 (6.8%)	105.62 (0.001 ***)	0.61
Weekly (last ten years)	women men	17 (6.1%) 24 (8.6%)		
Weekly (current)	women men	11 (3.9%) 24 (8.6%)		
Daily (last ten years)	women men	3 (1.1%) 4 (1.4%)		
	women men	1 (0.4%) 9 (3.2%)		
Daily (present)	women men	2 (0.7%) 3 (1.1%)		
	women men	1 (0.4%) 1 (0.4%)		
Several times a day (last ten years)	women men	72 (25.6%) 1 (0.4%)		
Several times a day (present)	women men	142 (50.3%) 6 (2.1%)		

Note: *** *p* < 0.001.

**Table 3:** The frequency with which pornography has been watched in the last ten years as a function of age.

Frequency of Pornography Watching	Age	*n* (%)	*x^2^* (*p*)	*Vcramer*
Sometimes (last 10 years)	18–21 <22	121 (43.2%) 30 (10.7%)	12.45 (0.014 *)	0.21
Sometimes (current)	18–21 <22	66 (23.5%) 19 (6.8%)	10.84 (0.028 *)	0.20
Weekly (last 10 years)	18–21 <22	27 (9.6%) 14 (5%)		
Weekly (current)	18–21 <22	26 (9.3%) 9 (3.2%)		
Daily (last 10 years)	18–21 <22	3 (1.1%) 4 (1.4%)		
	18–21 <22	5 (1.8%) 5 (1.8%)		
Daily (current)	18–21 <22	2 (0.7%) 3 (1.1%)		
	18–21 <22	1 (0.4%) 1 (0.4%)		
Several times a day (last ten years)	18–21 <22	65 (23.3%) 11 (3.9%)		
Several times a day (current)	18–21 <22	123 (43.9%) 25 (8.9%)		

Note: * *p* < 0.05.

**Table 4:** Positive effects that are valued most as a function of gender.

Positive Effects of Pornography	Gender	*n* (%)	*x*^2^ (*p*)	*Vcramer*
To satisfy curiosity (last ten years)	women men	73 (26.1%) 30 (10.7%)	50.66 (0.001 ***)	0.43
To satisfy curiosity (currently)	women men	14 (5%) 11 (3.9%)	58.55 (0.001 ***)	0.46
Learning about sex (last 10 years)	women men	9 (3.2%) 5 (1.8%)		
Learning about sex (currently)	women men	6 (2.1%) 5 (1.8%)		
Masturbating to your heart’s content (last ten years)	women men	22 (7.9%) 25 (8.9%)		
	women men	31 (11.1%) 43 (15.4%)		
Masturbating to your heart’s content currently)	women men	58 (20.7%) 1 (0.4%)		
	women men	- 1 (0.4%)		
Relationship with friends (last ten years)	women men	54 (19.3%) 1 (0.4%)		
Relationship with friends (currently)	women men	41 (14.6%) 4 (1.4%)		
No response (last ten years)	women men	60 (21.4%) 2 (0.8%)		
No response (currently)	women men	117 (41.7%) 7 (2.5%)		

Note: *** *p* < 0.001; -: not applicable.

**Table 5:** Current Negative Effects according to Gender.

Negative Effects of Pornography	Gender	*n* (%)	x^2^ (*p*)	*Vcramer*
Spent too much time (last ten years)	women men	35 (12.5%) 17 (6.1%)	36.88 (0.001 ***)	0.36
Spent too much time (currently)	women men	19 (6.8%) 12 (4.3%)	50.06 (0.001 ***)	0.42
Affected my relationship with my partner (last ten years)	women men	7 (2.5%) 2 (0.7%)		
Affected my relationship with my partner (currently)	women men	129 (46.1%) 8 (2.9%)		
Spent too much time (last ten years)	women men	- 2 (0.7%)		
	women men	0 (0%) 3 (1.1%)		
Spent too much time (currently)	women men	54 (19.3%) 28 (10%)		
	women men	51 (18.2%) 31 (11.1%)		
No negative effect (last ten years)	women men	144 (44.6%) 10 (3.6%)		
No negative effect (currently)	women men	22 (7.9%) 5 (1.8%)		

Note: *** *p* < 0.001; -: not applicable.

**Table 6:** Evaluation of Porn Addiction according to Gender.

Porn Addiction	Gender	*n* (%)	*x*^2^ (*p*)	*Vcramer*
Not at all (last ten years)	women men	171 (61.1%) 39 (13.9%)	38.33 (0.001 ***)	0.37
Not at all (currently)	women men	146 (52.1%) 45 (16.1%)	34.77 (0.001 ***)	0.35
A little (last ten years)	women men	8 (2.9%) 16 (5.7%)		
A little (currently)	women men	8 (2.9%) 10 (3.6%)		
Yes, it is possible (last ten years)	women men	2 (0.7%) 3 (1.1%)		
	women men	- 2(0.7%)		
Yes, it is possible (currently)	women men	39 (13.9%) 2 (0.7%)		
	women men	67 (23.9%) 2 (0.7%)		

Note: *** *p* < 0.001; -: not applicable.

## Data Availability

The data are available through the corresponding author.
